# Risky Behaviors Among Ohio Appalachian Adults

**Published:** 2006-09-15

**Authors:** Mary Ellen Wewers, M Katz, E D Paskett, Darla Fickle

**Affiliations:** School of Public Health, The Ohio State University; The Ohio State University School of Public Health, Columbus, Ohio, and The Ohio State University Comprehensive Cancer Center, Columbus, Ohio; The Ohio State University School of Public Health, Columbus, Ohio, and The Ohio State University Comprehensive Cancer Center, Columbus, Ohio; The Ohio State University Comprehensive Cancer Center, Columbus, Ohio

## Abstract

This article describes the prevalence of risky behaviors known to be associated with increased cancer morbidity and mortality among Ohio Appalachian adults. These behaviors, or risk factors, include: 1) tobacco use; 2) energy imbalance (involving poor diet, obesity, and physical inactivity); and 3) sexual behaviors. We report current estimates of the prevalence of these behaviors among Ohio Appalachian adult residents and review social, psychological, and biological variables associated with these risky behaviors. We also present recent empirical studies that have been completed or are in progress in Ohio Appalachia. Finally, we discuss how these studies help bridge well-documented gaps in the literature.

## Introduction

Disparities in cancer incidence have been recently reported in rural regions of Appalachia that include Kentucky, Pennsylvania, and West Virginia (1). Similar disparities are evident in Ohio Appalachia for all cancer sites and types combined, and there is a similar pattern in cancer mortality rates among Appalachian residents of Ohio compared with their non-Appalachian counterparts (2,3). Several factors may be responsible for these disparities, including increased prevalence of risky behaviors, lack of preventive cancer care, and limited access to health care. The purposes of this article are to 1) briefly describe the characteristics of Ohio Appalachia; 2) report the prevalence of risky behaviors in Ohio Appalachia known to be associated with cancer (i.e., tobacco use; obesity, poor diet and physical inactivity; and sexual behaviors); 3) identify social, behavioral, and biological factors that contribute to these risky practices; and 4) present relevant examples of recently completed and ongoing research in Ohio Appalachia. Finally, we offer recommendations for further research.

## Ohio Appalachia

The Appalachian region of Ohio includes 29 of the state's total 88 counties, or 33% of the state in square miles, and accounts for 12% of Ohio's total population (4). Compared with other parts of Ohio, the Appalachian region has been characterized by low socioeconomic status (SES), including lower household incomes, higher poverty rates, less education, and lower-paying occupations (4). The 20 Ohio counties with the lowest median household incomes were all in Appalachia, as were 19 of the 20 Ohio counties with the highest poverty rates. Appalachian children are 25% more likely to be raised in poor households. Twenty-two percent of Ohio Appalachian residents have no high school diploma, compared with 16% of residents in Ohio's non-Appalachian region. Four of the 29 Appalachian Ohio counties are classified as "distressed," and another six counties are classified as "at risk" (4). Distressed counties are considered to be the most economically depressed, while at-risk counties have a higher probability of becoming economically distressed. 

Ohio Appalachian residents have logistical barriers to overcome in accessing health care. With only 32 registered hospitals and 1723 physicians for more than 1.4 million residents, Ohio Appalachian residents are more likely than other Ohioans to travel greater distances to obtain health care, and they do so without the infrastructure of a public transportation system (5). The risk of being uninsured among Ohio residents is highest in the Appalachian counties (5). There are nearly 200,000 uninsured Ohio Appalachian residents; 14.7% of adults and 6.3% of children lack health insurance. Approximately 65% of the 29 Appalachian counties in Ohio are designated as either geographic or specific population health professional shortage areas (5). These disparities in health care access represent a significant burden, often leading to important differences in health status.

## Background and Significance of Risky Behaviors in Ohio Appalachia

### Tobacco use

Tobacco use is the single most preventable cause of premature morbidity and mortality (6), and the behavior is increasingly prevalent among poor and medically underserved groups. Smoking behavior has a significant impact on health inequalities and specific diseases (7). It is anticipated that mortality rates due to tobacco-attributable diseases will be progressively more apparent among this at-risk, vulnerable group (8). Cigarette smoking is a risk factor for many cancers, including cancer of the lung, bladder, head and neck, pancreas, and cervix. Approximately one third of all cancer mortality is due to lung cancer. An association between a smoking behavior and other life-threatening conditions, such as cervical cancer, has also been described in past epidemiological investigations (9). As such, interventions that promote permanent smoking cessation have the potential to reduce preventable diseases.

An estimated 44.5 million people, or 20.9% of the U.S. adult population, smoke (10). Sociodemographic characteristics indicate that 23.4% of men and 18.5% of women currently smoke. Smoking prevalence is highest for those aged 18 to 24 years (23.6%) and 25 to 44 years (23.8%). Smoking prevalence also differs according to SES. Prevalence is highest for individuals living below the poverty line (29.1%) and for individuals with less than a high school diploma (34%). Thirty-nine percent of those with a General Educational Development (GED) diploma are categorized as current smokers. Appalachians demonstrate a higher prevalence of tobacco use than the U.S. population in general (3,10). Between 1999 and 2003, the proportion of current smokers in Ohio Appalachian counties was 31.5% compared with 26.1% in non-Appalachian Ohio counties (3).

The factors responsible for an increased prevalence of tobacco use in Ohio Appalachia remain poorly understood. There is a growing body of evidence confirming that tobacco use occurs in greater proportions among certain groups within the United States, including Ohio Appalachia. Those who are poor and less educated have higher rates of smoking than their more privileged counterparts. As such, they also suffer tobacco-attributable morbidity and mortality at significantly higher rates. Women who smoke, like men, are at increased risk of cancer, heart disease, and lung disease (11). Smoking prevalence is higher among women of reproductive age (15–44 years) (21.5%) than among the overall population of adult women (18.5%) (10). Population-based estimates of continued smoking throughout pregnancy are 18% (11). SES as indicated by education level has a dramatic effect on prenatal smoking behavior. In 1998, 25.5% of women with less than a high school education smoked throughout pregnancy compared with only 2.2% of mothers with a college degree.

Behavioral and biological factors also play a role in tobacco use. There is compelling psychopharmacological evidence to explain maintenance of smoking behavior because of nicotine dependence (12). Poorer smokers are more nicotine dependent (7), suggesting that social structure may influence the continuance of smoking; however, investigations of the mechanisms responsible for increased dependence among poorer smokers are limited. Engaging in a tobacco use behavior has been referred to as *self-medication*, primarily because of the reinforcing effects associated with nicotine (7). These effects include stress reduction, relaxation, and euphoria. Because poorer individuals often experience increased stressors, smoking may serve as an effective strategy for managing the unpleasant symptoms associated with stress.

### Energy imbalance

Energy balance is the complex interaction of diet, physical activity, and genetics on growth and body weight over an individual's lifetime. Today, nearly two thirds of the U.S. adult population is overweight or obese. Being overweight or obese and sedentary are associated with increased risk for cancer, other major chronic diseases, and death (13). A diet high in fruits, vegetables, and grains and low in saturated fat, red meat, or other animal fat and regular physical activity play a role in the primary prevention of cancer (14).

According to the 2003 Ohio Behavioral Risk Factor Surveillance System, only 73.6% of Ohio residents participated in any type of physical activity during the past month, compared with the nation's rate of 76.9% (15). Consumption of fruits and vegetables five or more times per day by Ohio residents was 22.7%, which is similar to the nation's rate of 22.6% (15). Further evaluation of the Ohio cancer-related health behavior prevalence rates distinguish between residents of Appalachian and non-Appalachian Ohio. Adults aged 18 years and older living in Appalachia have higher rates of obesity than adult residents of non-Appalachian Ohio counties (23.4% compared with 22.3%) and higher rates of no reported leisure-time physical activity (29.4% compared with 28.6%) (3). Adults in Appalachia also have higher rates of inadequate (less than five servings per day) fruit and vegetable consumption (78.9%) than adults in non-Appalachian Ohio counties (77.1%) (3). The indirect effect of these data may explain why residents of Ohio Appalachia were more likely to report their health as *poor* or *fair* (18.3%) than were residents of urban, suburban, or rural non-Appalachian counties (13.4%–15.9%) (3). 

At the individual level, obesity is associated with lower education, lower SES, and being a member of a minority population (16). At the environmental level, obesity rates and physical inactivity are higher in low SES neighborhoods (17). In a nationally representative cohort, low-SES census-block groups were less likely to have access to recreational facilities, which in turn was associated with decreased physical activity and increased overweight (18). 

There is growing evidence that obesity and SES may be related to dietary energy density and energy costs (19). Energy-dense diets have a lower satiating power and may result in passive overconsumption and, thus, weight gain (20). Craving of energy-dense fats and sweets has been explained by neurotransmitter imbalance (21), and excess consumption of sweets has been explained by addictive personality, stress, and depression. The lack of access to healthy foods (22) and the higher cost of these foods (23) suggest that strategies to help low SES populations change to healthier diets may be difficult without changes in public policy.

### Risky sexual behaviors

The development of cervical cancer depends on a variety of social and behavioral factors acting together with biological factors, such as high-risk human papillomavirus (HPV) variants. The residents of Ohio Appalachia are relatively isolated geographically because of topographical boundaries created by hills and valleys. Although this isolation has led to self-reliant communities, it may also contribute to the high prevalence of risky behaviors, including risky sexual behaviors. Risky sexual behaviors for cervical cancer have been described as 1) engaging in sexual intercourse at an earlier age than 18 years, 2) having more than two sexual partners, 3) having a history of being treated for a sexually transmitted infection, or 4) having a current or past sexual partner who has been treated for a sexually transmitted infection (24).

Risky sexual behaviors are influenced by determinants such as acceptance of risky sexual behavior, access to health care, SES, and cultural or physical environment. Little is known about the cultural or physical environmental influences in Appalachia on risky sexual behaviors. Cultural norms related to casual sexual relations may be important to determine, as extramarital sexual relationships have been found to be associated with HPV positivity (25). Unfortunately, the lack of definitive evidence about factors such as screening, incidence and stage, health care service delivery, resources (both monetary and nonmonetary), and acceptance of interventions by the target population (e.g., rural Appalachian women) have served as barriers to our understanding about individuals at risk for HPV and cervical cancer (26). Many counties in Appalachia do not have any physicians or have far fewer physicians than the national average (27). Data from the Ohio Cancer Incidence Surveillance System (3) demonstrate an inverse association between census tract income level and cervical cancer incidence.

Risky sexual behaviors place women at high risk for acquiring HPV, the primary etiologic agent in cervical cancer (24,28). Persistent cervical infection with certain types of HPV is the single most important risk factor for cervical cancer. Infection with HPV type 16 accounts for more than 50% of cervical cancers and high-grade dysplasia; infection with HPV of types 16, 18, 31, and 45 accounts for 80% of cervical cancers (29). HPV may also play a role in cancers of the anus, vagina, oropharynx, and penis (30).

Prevalence data on risky sexual behaviors in Ohio Appalachia are not readily available. Data on rates of sexually-transmitted infections do not indicate differences between rural and nonrural populations, and there are no data on HPV rates in rural or Appalachian populations. Compared with the median of all female students in the 2003 Youth Risk Behavioral Surveillance System (YRBSS), more female high school students in West Virginia reported having had sexual intercourse (WVa 54.9% vs 43.6% national), having had sexual intercourse before age 13 (WVa 4.3% vs 3.5% national), having had sexual intercourse with four or more partners (WVa 16.6% vs 11.6% national), or having had sexual intercourse with one or more partners within the last 3 months (WVa 44.0% vs 34.1% national) (31). HPV is likely the most common sexually-transmitted disease among young, sexually active people and is of increasing public health importance. Because all of West Virginia is designated as Appalachia, it might be assumed that these data indirectly suggest that women in Appalachia may be at greater risk for contracting HPV and subsequently at higher risk for cervical cancer. 

Factors associated with cervical cancer are not limited to risky sexual behaviors, but the sexual behaviors listed above may elevate the likelihood for cervical disease. The risky sexual behaviors of women living in Ohio Appalachia may be influenced by family-related demographics, neighborhood, or by the culture of Ohio Appalachia. Risky sexual behaviors, influenced by geographic isolation and local culture and norms, put women at increased risk for acquiring HPV.

## Examples of Risky Behavior Research Studies in Ohio Appalachia

### Tobacco risk and counseling

Rural adult Ohio Appalachian residents have participated in a smoking cessation study entitled Tobacco Risk and Counseling (TRAC). The study was approved by The Ohio State University's institutional review board. This project was jointly designed by researchers and community residents from two Ohio Appalachian counties. The project described tobacco consumption variables among rural adult Appalachian tobacco users (32). Participants aged 18 years and older (n = 249) were enrolled at summer fairs in the two rural Ohio Appalachian counties in a face-to-face interview about tobacco consumption variables and knowledge of the health effects of tobacco. The interviews were administered by community residents. The majority of participants were categorized as precontemplators (not interested in quitting smoking in the next 6 months), although 21% were categorized as in the preparation stage of change (interested in quitting smoking in the next 30 days). Mean age of smoking initiation was 16.6 years, and number of cigarettes smoked per day was significantly higher for men than for women. One third of the men reported the use of smokeless tobacco. The majority had not tried to quit for more than a year, and the average number of previous quit attempts was low. One half of participants had been advised to quit by their physician. Few had used nicotine replacement with past quit attempts, but more than half would consider this approach with future attempts. Knowledge about the health effects of smoking indicated that most participants were aware of the relationship between smoking and cancer, but less than one half recognized its association with heart disease. Participants with less education were less informed about the health effects of smoking to self and nonsmokers.

Qualitative information was obtained through four focus groups (with former and current tobacco users) and indicated that nicotine addiction was a major theme for engaging in the behavior (33). Dependence was significantly associated with barriers to quitting. Clearly, the addictive nature of nicotine and its pharmacological aspects indicated a need for pharmacotherapy to be incorporated into tobacco treatment plans. Also, the importance of family support and personal independence in relation to tobacco use were evident. Common reasons for quitting smoking were personal health, expense, exposure of others to secondhand smoke, and desire to be a good example for others, especially younger family members. Participants identified reliance on family members for support as among the helpful strategies for smoking cessation. The groups agreed that a lay adviser (i.e., an individual who represented the values of the Appalachian community and was viewed as credible) could serve as an effective facilitator of a cessation intervention.

Information about tobacco use characteristics and apparent themes for continued use, as well as reasons for quitting, should assist in the generation of additional hypotheses to be tested among Appalachian smokers. These factors may assist researchers and community leaders in the design and testing of prevention and cessation interventions that are specifically tailored to Appalachian tobacco users.

### Center for Population Health and Health Disparities

A multidisciplinary group of investigators from The Ohio State University and the University of Michigan received funding in 2003 to establish a Center for Population Health and Health Disparities. The Center will initially focus on the goal of understanding *why* there are high rates of cervical cancer and deaths from cervical cancer in Ohio Appalachia.

The Center will conduct three related projects in 14 clinics that represent the general population of women aged 18 and older in the Appalachian region and enlist the help of an internal and an external advisory committee. Community partners organized into a community advisory board and a consortium of community organizations have been established to facilitate the accomplishment of project goals. The goal of the first project is to increase early detection of cervical cancer by increasing the proportion of Appalachian women who receive Papanicolaou (Pap) tests at appropriate intervals and return for follow-up when necessary. The second project will test the effectiveness of an intervention facilitated by a lay health educator for women who smoke. The intervention will promote smoking cessation and validate cessation with saliva cotinine measurements. The third project will examine the social, behavioral, and biological variables that contribute to an increased risk of an abnormal Pap test among Appalachian women. (More information about these projects is available from http://sph.osu.edu/divisions/abouttheschool/diversity/cervical.)

### Amish Cancer-related Lifestyle Project

The Amish living in the Holmes County area of Ohio Appalachia have lower overall cancer incidence rates (34). To evaluate the role that lifestyle factors may play in the low cancer incidence rates, an interdisciplinary team of researchers was established at The Ohio State University. The Amish Cancer-related Lifestyle Project uses community-based participatory research methods and is in a partnership with the Amish communities of Ohio Appalachia (35). The study was approved by the university's institutional review board.

The Amish are a unique population living in Ohio Appalachia; they have lifestyle factors that are different from those of the general U.S. population (36,37). To examine the differences in lifestyle factors and to assess whether differences in lifestyle may contribute to the lower cancer incidence rates, face-to-face in-depth interviews were conducted among Amish adults living in Ohio Appalachia. 

To gain acceptance by this population so that interviews about lifestyle factors could be conducted, the research team established a partnership with members of the Amish community. Several lectures and discussions with a focus on cancer and family history were held in different church districts to establish a relationship with the Amish community. A study of family history and cancer was conducted among a random sample of 92 Amish households in the Holmes County, Ohio, region. The sample was taken from the 1996 Ohio Amish Directory. Members of the research team attend regional Amish leadership meetings to provide results to the community and to discuss potential new research in the Amish community. This exchange has allowed trust to be established between the key stakeholders of the Amish community and additional members of The Ohio State University cancer research team. This trust has led to the development of a partnership focused on the cancer-related issues of the Amish community.

A questionnaire focusing on lifestyle behaviors was developed, and key informants from the Amish community reviewed the questionnaire for cultural acceptance. Several small changes were made after pilot testing the questionnaire among several Amish adults.

The sample for this study was the original random sample of 92 households from the cancer family history study. If individuals moved, an attempt was made to locate the original head of household. If the household members were no longer Amish or refused to participate, replacement homes were randomly sampled from the same church district. A letter of introduction was mailed to each household, and a member of the research team trained in interviewing techniques and aware of Amish culture visited each home to arrange a date and time for the interview. Each interview lasted approximately 2 hours. The questionnaire covered demographic characteristics, general health and use of conventional and alternative medical services, personal and family cancer and medical history, cancer screening test use, medications and supplement use, tobacco and alcohol use, health literacy, physical activity and work history, and nutrition and food storage. Additionally, the participant's height and weight were measured at the visit. Each participant was asked to provide a saliva sample for cotinine testing to verify smoking status and was asked to wear a pedometer for one week so that physical activity levels could be monitored objectively by steps per day.

The response rate was 66% (75 homes) and included 134 completed interviews (72 women and 62 men). Approximately 60% of those interviewed lived in the same Ohio county their entire lives. During the ongoing research process, the research team members have participated in several events to maintain a productive partnership with Amish community members. The researchers have presented study updates at several Amish leadership meetings, have participated in county-level Amish health fairs, and have continued to communicate with study participants. The researchers plan to provide the Amish community with the results of the project. As an outgrowth of this collaboration, the researchers will work with the Amish community to design and implement cancer prevention programs.

The Amish Cancer-related Lifestyle Project demonstrates how an individual's health is embedded within a social and physical environment. Rather than focusing on risk factors, this study focuses on lifestyle factors that may protect individuals from cancer. Strengths of this project are built upon the collaborative partnership developed between the Amish community and university researchers.

## Future Directions and Implications for Risky Behavior Research in Ohio Appalachia

Although there is an abundance of data about smoking and tobacco use across the United States, knowledge of tobacco use behaviors in Appalachia is limited to prevalence estimates. Understanding the social, behavioral, and biological dimensions of tobacco use among Ohio Appalachian women will assist in the development and testing of scientifically valid cessation interventions and may subsequently contribute to reduced risk of cancer among this vulnerable population. The role of social and contextual variables, such as the environment, neighborhood, community, and culture must be integrated into future investigations that attempt to understand the mechanisms responsible for tobacco use.

Inequality in the built environment and limited access to healthy food choices present challenges for behavioral interventions focusing on energy balance for residents of Ohio Appalachia. Future behavioral interventions to reduce cancer disparities must address these issues within the cultural context of Ohio Appalachia.

Additional efforts should be directed toward understanding what places Ohio Appalachian women at increased risk for cervical abnormalities and HPV infection. As noted, HPV is an important cause of cervical cancer. Better understanding of HPV prevalence rates in Appalachia and the impact of known risk factors, immune function, tobacco use, and diet on HPV infection is needed in this population. In addition, questions about differences in HPV virulence, prevalence types, oncogenic potential, transmission, reservoirs, and resistance in this population are important. This information is needed to begin intervention efforts focused on this topic in the Appalachian region of the United States. The projects of the Center for Population Health and Health Disparities are examples of community-based participatory research that is poised to answer questions associated with the increase in cervical cancer incidence and mortality among women living in Ohio Appalachia.
